# High-Density of FcγRIIIA^+^ (CD16^+^) Tumor-Associated Neutrophils in Metastases Improves the Therapeutic Response of Cetuximab in Metastatic Colorectal Cancer Patients, Independently of the HLA-E/CD94-NKG2A Axis

**DOI:** 10.3389/fonc.2021.684478

**Published:** 2021-06-15

**Authors:** Marie Denis Musquer, Nicolas Jouand, Morgane Pere, Juliette Eugène Lamer, Stéphane Bézieau, Tamara Matysiak, Roger Faroux, François-Xavier Caroli Bosc, Marie-Christine Rousselet, François Leclair, Jean-François Mosnier, Claire Toquet, Nadine Gervois, Céline Bossard

**Affiliations:** ^1^ Department of Pathology, University Hospital of Nantes, Nantes, France; ^2^ Université de Nantes, Inserm, CRCINA, Nantes, France; ^3^ LabEx IGO “Immunotherapy, Graft Oncology”, Nantes, France; ^4^ Biostatistics Plateform, University Hospital of Nantes, Nantes, France; ^5^ Department of Genetic, University Hospital of Nantes, Nantes, France; ^6^ Department of Gastroenterology, University Hospital of Nantes, Nantes, France; ^7^ Department of Gastroenterology, Hospital of La Roche sur Yon, La Roche sur Yon, France; ^8^ Department of Gastroenterology University Hospital of Angers, Angers, France; ^9^ Department of Pathology, University Hospital of Angers, Angers, France; ^10^ Department of Pathology, Hospital of La Roche sur Yon, La Roche sur Yon, France

**Keywords:** cetuximab, metastatic colorectal carcinoma, CD16, tumor-associated neutrophils, antibody-dependent cellular cytotoxicity, HLA-E/NKG2A axis

## Abstract

Antibody-dependent cellular cytotoxicity (ADCC) in the anti-tumor effect of cetuximab in metastatic colorectal cancer (mCRC) is only based on the impact of FcγRIIIA (CD16) polymorphisms as predictive of therapeutic response. However, nature, density and therapeutic impact of FcγRIIIA^+^ (CD16) effector cells in tumor remain poorly documented. Moreover, the inhibition of cetuximab-mediated ADCC induced by NK cells by the engagement of the new inhibitory CD94-NKG2A immune checkpoint has only been demonstrated *in vitro*. This multicentric study aimed to determine, on paired primary and metastatic tissue samples from a cohort of mCRC patients treated with cetuximab: 1) the nature and density of FcγRIIIA^+^ (CD16) immune cells, 2) the expression profile of HLA-E/β2m by tumor cells as well as the density of CD94^+^ immune cells and 3) their impact on both objective response to cetuximab and survival. We demonstrated that FcγRIIIA^+^ (CD16) intraepithelial immune cells mainly correspond to tumor-associated neutrophils (TAN), and their high density in metastases was significantly associated with a better response to cetuximab, independently of the expression of the CD94/NKG2A inhibitory immune checkpoint. However, HLA-E/β2m, preferentially overexpressed in metastases compared with primary tumors and associated with CD94^+^ tumor infiltrating lymphocytes (TILs), was associated with a poor overall survival. Altogether, these results strongly support the use of bispecific antibodies directed against both EGFR and FcγRIIIA (CD16) in mCRC patients, to boost cetuximab-mediated ADCC in *RAS* wild-type mCRC patients. The preferential overexpression of HLA-E/β2m in metastases, associated with CD94^+^ TILs and responsible for a poor prognosis, provides convincing arguments to inhibit this new immune checkpoint with monalizumab, a humanized anti-NKG2A antibody, in combination with anti- FcγRIIIA/EGFR bispecific antibodies as a promising therapeutic perspective in *RAS* wild-type mCRC patients.

## Introduction

The introduction of targeted therapies, especially monoclonal antibodies targeting epidermal growth factor receptor (EGFR), such as cetuximab, a human-mouse chimeric IgG1 antibody, in the treatment of patients with metastatic colorectal cancer (mCRC), has led to a consistent improvement in survival. *RAS* mutations are the only well-established predictive biomarkers of resistance to cetuximab (1). Antibody-dependent cellular cytotoxicity (ADCC) mechanism by which Fc region of the antibody binds to the Fc gamma receptors expressed by immune cells plays a key role in the antitumor effect of IgG1 antibodies (2), and *in vitro* studies demonstrated that cetuximab can mediate ADCC against several tumor cell lines (3). Fcγ receptor type IIIA (FcγRIIIA), commonly expressed on NK cells as well as on several other immune cells including γδ T cells, neutrophils and monocytes/macrophages, is well known for its role in ADCC (4, 5). More specifically, the impact of FcγRIIIA polymorphisms as a predictive factor for CRC patients treated with cetuximab suggests an important role of ADCC in cetuximab efficacy (6, 7).

In parallel, some *in vitro* data based on CRC cell line demonstrated that the non-classical major histocompability complex (MHC) Class I, human leucocyte antigen E (HLA-E) expressed by tumor cells and stabilized by β2 microglobulin (β2m) co-expression, inhibits the cetuximab-mediated cellular cytotoxicity induced by natural killer (NK) cells. Indeed, Levy et al. specified that this inhibitory effect on ADCC was dependent of the direct interaction between HLA-E/β2m, overexpressed by tumor cells, and its specific inhibitory receptor, CD94/NKG2A (NK group 2 member A) expressed by NK cells (8). HLA-E is a poorly polymorphic human MHC class Ib molecule, ubiquitously expressed at low levels on the cell surface of most tissues, whose functional expression requires its stabilization by the light chain β2-microglobulin (β2m) to form a membrane heterodimeric ligand. This ligand, aberrantly overexpressed by tumor cells in several solid tumors, interacts either with the inhibitory CD94/NKG2A receptor, or the activating CD94/NKG2C (NK group 2 member C) receptor selectively expressed by immune cells with cytolytic functions such as NK cells, NKT (natural killer T) cells and a subset of T cells (9). Indeed, we have previously shown that overexpression of HLA-E/β2m by tumor cells (primary tumors) characterizes a subgroup of 23% of CRC. In the tumor microenvironment of those primary HLA-E/β2m^+^ tumors, NK cells and mostly CD8^+^ αβ T lymphocytes preferentially expressed the inhibitory CD94/NKG2A receptor. These two immune markers (HLA-E/β2m and CD94/NKG2A) were associated with poor prognosis (10, 11). Besides, the CD94/NKG2A receptor, recently considered as a new inhibitory immune checkpoint that blocks the terminal cytolytic tumor-attack in the tumor microenvironment, can be blocked by the anti-NKG2A antibody, monalizumab, a therapy associated with promising clinical responses in gynecologic and head and neck refractory cancers (12).

Altogether, these results prompted us to address for the first time in a cohort of *RAS* wild-type mCRC patients treated with cetuximab, on paired primary and metastatic tissue samples: 1) the nature and density of FcγRIIIA^+^ (CD16) immune cells, 2) the expression profile of HLA-E/β2m by tumor cells as well as the density of intra-tumor CD94^+^ immune cells and 3) their impact on both objective response to cetuximab and overall survival.

## Patients and Methods

### Patients and Tissue Samples

Forty-three patients treated with cetuximab for a mCRC at the “Centre Hospitalier Universitaire” (CHU) of Nantes (n=13), Angers (n=3) and La Roche sur Yon (n=7), and at the “Hôpital du Confluent” of Nantes (n= 20) between 2000 and 2015, were retrospectively included in this study. For these patients, tissues samples of both primary and matched metastatic tumors were available. Rectal carcinomas previously treated with chemo-radiotherapy were excluded from the study, as well as patients with unavailable tissue samples for immunohistochemistry and/or complementary molecular analyses. As the patients were treated before the cetuximab new indications in July 2013, only the *KRAS* codon 12/13 *(*exon 2) wild-type status was known at the time of the study. For the purpose of this study, all primary and metastatic collected tissues were re-analyzed for other *RAS* mutations in four additional *KRAS* codons (exons 3 and 4) and six *NRAS* codons (exons 2, 3, and 4) using HRM PCR and sequencing. Additional mutations were found in 4 patients (two 436G>A mutations in *KRAS* exon 4 in both primary tumor and paired metastasis, one 351A>C mutation in *KRAS* exon 4 in a primary tumor and one 181C>A mutation in *NRAS* exon 3 in a metastasis), thus resulting in 39 patients with a wild-type *RAS* status eligible for the current study. All patients received Cetuximab plus chemotherapy (or other cytotoxic agent) for their metastatic disease (Irinotecan n=18; FOLFIRI regimen (irinotecan + folinic acid + 5 fluorouracil combination) n=10; FOLFOX regimen (oxaliplatin + folinic acid + 5 fluorouracil combination), n=6; Camptomycine n=2, Afatinib n=1), except two patients who received Cetuximab alone. Distant metastases were defined as synchronous or metachronous according to their identification, at the time of the primary tumor diagnosis or more than 12 months after, respectively (13).

For each patient, formalin-fixed and paraffin-embedded tissues representative of the primary tumor (37 surgical resections and 2 biopsies) and matched metastasis (26 surgical resections and 13 biopsies) were selected. Tissue samples were collected from the archives of the Department of Pathology or of each participating center, and were processed according to the guidelines of our institution and of the French Ethics Committee for Research on human tissues. The institutional board of the University Hospital of Nantes approved this study. Our tissue biocollection has been registered with the French Ministry for Higher Education and Research (DC-2014-2206) with approval from the ethic committee (CPP Ouest IV-Nantes). Our study was conducted in accordance with the Helsinki Declaration.

For the flow cytometry analysis, as no viable fresh tissues samples were available for our retrospective cohort of 39 patients, we included prospectively four successive patients who benefited from a colectomy for a CRC at the University Hospital of Nantes, and who did not receive any neoadjuvant chemotherapy or radiotherapy. A fresh tissue sample of the primary tumor was immediately dissected for flow cytometry analyses. Each patient included signed an informed consent form.

### Clinical Evaluation and Response Criteria

Tumor response was assessed 12 weeks after starting chemotherapy by computed tomography (CT-Scan) according to the Response Evaluation Criteria in Solid Tumors (RECIST) criteria and classified as partial response (PR), stable disease (SD) or progression disease (PD). Patients with SD or PD were defined as non-responders (14). Overall survival (OS) was measured from the date of first diagnosis (tumor resection or biopsy) to the date of death related to CRC or to the latest follow-up, and censured at five years.

### Tumor Sample Dissociation

In order to precisely define the nature of FcγRIIIA^+^ (CD16) immune cells in the tumor microenvironment of CRC, we assessed by flow cytometry the phenotype of FcγRIIIA^+^ (CD16) cells from cell suspensions derived from four fresh primary tumors of CRC patients included prospectively. Fresh dissected tumor samples were treated as described previously (15). Briefly, the tissue fragments were cut into small pieces (1–2 mm^2^) at room temperature in RPMI 1640 medium (Life Technologies), and then transferred into a GentleMACS C tube (MiltenyiBiotec) for two rounds of non-enzymatic mechanical dissociation with a GentleMacsOctoDissociator (Program A.01, MiltenyiBiotec). The resulting cell suspension was filtered through a 40-µm cell strainer (Dutscher), and then centrifuged for 15 min at 600*g* at room temperature, and finally resuspended in RPMI 1640 before the *ex-vivo* flow cytometry analyses.

### Flow Cytometry Analyses

**Figure 1:**
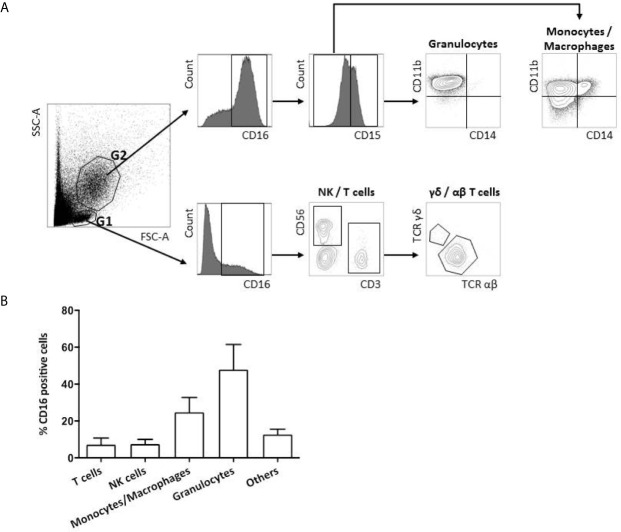
FcγRIIIA (CD16) expression on tumor-infiltrating immune cells. Flow cytometry gating strategy of FcγRIIIA (CD16) expression on a representative primary tumor sample from a CRC patient **(A)**. FcγRIIIA (CD16) positive cells are gated among the living lymphoid (G1) and myeloid (G2) cells and different immune sub-populations are highlighted according to their phenotype: T cells (CD3^+^TCRαβ^+^ or γδ^+^), NK cells (CD3^−^CD56^+^), monocytes/macrophages (CD15^−^CD11b^+^CD14^−/low^) and granulocytes (CD15^+^CD11b^+^CD14^−^). Frequency of FcγRIIIA (CD16) positive infiltrating immune sub-populations among total FcγRIIIA^+^ (CD16) positive living cells from the 4 non metastatic CRC patients included prospectively. The bar charts represent mean +/- standard deviation **(B)**.

Immediately after fresh tumor samples dissociation, 2 × 10^5^ cells were incubated in 100 µl of brilliant stain buffer (BD Biosciences) with one of the three following combinations of antibodies at saturating concentrations at 4°C for 30 min. To identify FcγRIIIA^+^ (CD16) expressing cells in the lymphoid and myeloid tumor-infiltrating cells, the CD16-Phycoerythrin (PE) mAb (clone 3G8, BD Pharmingen) was used in association with three different combinations of antibodies: 1) CD3-Brillant Violet (BV) 421 (clone UCHT1, BD Pharmingen), TCR αβ-BV510 (clone T10B9.1A-31, BD Pharmingen), TCR γδ-Allophycocyanin (APC) (clone B1, BD Pharmingen), CD8α-BV650 (clone RPA-T8, BD Pharmingen), CD4-BV786 (clone L200, BD Biosciences); 2) CD3-BV421 (clone UCHT1, BD Pharmingen), CD56-APC (clone B159, BD Pharmingen), TCRα24-BV711 (clone 6B11, BD Biosciences); and 3) CD15-APC (clone HI98/HIM1, BD Pharmingen), CD11β-BV510 (clone ICRF44/44, BD Pharmingen), CD14-PE-Cyanin 7 (clone M5E2, BD Biosciences). Dead cells were identified with the viability marker Zombie NIR (BioLegend). Following incubation, cells were washed twice in PBS with 0.1% BSA medium and finally suspended in 200 µl of PBS. Stained cells were analyzed by flow cytometry using a LSRII flow cytometer and BD Diva software (BD Biosciences). The compensation adjustments were made using the anti-mouse Ig, k/negative control compensation particle set (BD Biosciences). Flow cytometry gating strategy is depicted in **Figure 1A**. FcγRIIIA^+^ (CD16) immune cells were gated in lymphoid (G1) and myeloid (G2) living cells among the lymphoid cells, NK cells and T cells were identified by their CD3^−^CD56^+^ or CD3^+^TCR αβ/TCR γδ expression profile, respectively. Among the myeloid cells, granulocytes and monocytes/macrophages were identified by their CD15^+^CD11b^+^ CD14^−^ or CD15^−^CD11b^+^CD14^+/−^ phenotype.

### Immunohistochemical Analyses

We analyzed by immunohistochemistry, both in primary tumors and paired metastasis for each patient, the density of intraepithelial FcγRIIIA^+^ (CD16) immune cells, as immune cells in close contact with tumor cells can be considered as the most efficient effector cells to kill tumor cells *via* ADCC. Immunohistochemistry was performed on full tissue sections of all tumors (except for CD94/NKG2 and NKp46 performed on 19 cases due to exhaustion of some paraffin blocks). Slides were stained with the following primary antibodies: CD16 (clone 2H7, 1:100, Leica), NKp46 (clone 195314, 1:100, R&D Systems), CD8 (clone C8/144B, 1:50, Dako), CD163 (clone 10D6, 1:100, Leica) HLA-E (clone MEM-E/02, dilution 1:200, Serotec), β2m (clone SC11.1, 1:200, gift of J Gachet U1232, Nantes), and CD94/NKG2 (clone B-D49, 1:20, Diaclone). The immunological reactions were visualized with the Envision detection system (DakoCytomation) and 3,3-diaminobenzidine tetrahydrochloride as the chromogen. The slides were counterstained with Mayer’s hematoxylin. The percentage of intraepithelial immune cells expressing CD8, FcγRIIIA (CD16), CD94/NKG2 or NKp46, was assessed by counting the number of labeled cells per 100 tumor cells on three fields at a magnification of 400.

The level of HLA-E expression, assessed by staining intensity, was compared with the paired normal colonic mucosa, as previously described (10). Furthermore, since a heterogeneous expression of HLA-E by tumor cells in whole tissue sections was observed, the percentage of strongly positive HLA-E tumor cells was also taken into account. Accordingly, HLA-E overexpression by tumor cells was defined as more than 5% of tumor cells showing a strong staining compared with that of the normal colonic mucosa.

### Statistics

Clinical and pathological data, as well as the percentages of CD8^+^, FcγRIIIA^+^ (CD16), NKp46^+^ or CD94^+^, and HLA-E/β2m overexpression by tumor cells, were entered into a database. Mann-Whitney test was used to test the relationship between the density of the different intraepithelial immune cells (CD8, NKp46, FcγRIIIA (CD16), CD94) and the tissue localization (normal colonic mucosa, primary tumor and metastases). Associations between the different immunological markers (CD8, FcγRIIIA (CD16), CD94 and HLA-E/β2m) and the objective response to cetuximab at 3 months were tested by Fisher’s test. Overall survivals were estimated using Kaplan-Meier method. The log-rank test and Cox regression analyses were used to assess survival differences between groups. For all analyses, a p value<0.05 was considered significant.

## Results

### Clinicopathological Features of Patients

The main clinicopathological features of the patients included in this study are summarized in **Table 1**.

**Table 1:** Clinicopathological features of mCRC patients treated with cetuximab in combination with chemotherapy.

Characteristics	Patients (n=39)
**Sex**	
Men	26 (67%)
Women	13 (33%)
**Age**	
Mean (range)	61,5 (44,6 - 78,1)
**Site of primary tumor**	
Right colon	8 (20%)
Left colon	23 (59%)
Transverse colon	4 (10%)
Rectum	3 (8%)
Colonic neobladder	1 (3%)
**Histological type**	
Adenocarcinoma, not otherwise specified	33 (85%)
Mucinous adenocarcinoma	6 (15%)
**pTNM stage**	
pT1	1 (3%)
pT2	0 (0%)
pT3	19 (49%)
pT4	19 (49%)
pNx	4 (10%)
pN0	4 (10%)
pN1	17 (44%)
pN2	14 (36%)
**Metastatic lesions**	
Synchronous	29 (74%)
Metachronous	10 (26%)
**Metastatic site**	
Liver	27 (69%)
Peritoneum	9 (23%)
Lung	3 (8%)
**Therapy in combination with cetuximab**	
FOLFOX regimen	6 (15%)
FOLFIRI regimen	10 (26%)
Irinotecan	18 (46%)
Camptomycine	2 (5%)
Afatinib	1 (3%)
**Best tumor response at 3 months**	
Partial response	15 (38%)
Stable disease	13 (34%)
Progression disease	11 (28%)

### FcγRIIIA^+^ (CD16) Tumor-Infiltrating Immune Cells Mainly Correspond to Myeloid and Lymphoid Cells

In order to precise the exact nature of tumor-infiltrating immune cells that express FcγRIIIA (CD16), a flow cytometry analysis was performed on fresh tumor tissues from surgically resected primary tumors of CRC. For this purpose, 4 non metastatic patients (1 man, 3 women; aged from 67.5 to 79 years; 2 right colon, 2 left colon; 4 adenocarcinoma NOS (3 stages II and 1 stage III) who do not receive any neoadjuvant chemotherapy or radiotherapy were included prospectively.

The nature and the absolute frequency of different FcγRIIIA^+^ (CD16) tumor-infiltrating cell types are summarized in **Table 2** and are illustrated in **Figure 1A**. The density of FcγRIIIA^+^ (CD16) cells in tumors, except 1 case, is quite homogeneous representing an average of 4% among all viable cells (immune and tumor cells). The case harboring a particularly rich population of FcγRIIIA^+^ (CD16) cells (92C—51%) corresponded to a very inflamed tumor with neutrophilic microabcess. In any case, those cells mainly corresponded to myeloid cells, i.e., granulocytes and macrophages (**Figure 1B**). Indeed, about 48% and 25% of FcγRIIIA^+^ (CD16) immune cells featured a granulocyte-like (CD15^+^CD11b^+^CD14^−^) or a monocyte/macrophage-like phenotype (CD15^−^CD11b^+^CD14^+/−^), respectively. Furthermore, we also identified FcγRIIIA^+^ (CD16) lymphoid cells representing NK cells (3.2%–14.8%, mean 7.5%) and T lymphocytes (1.5%-16.5%, mean 7.2%), mainly corresponding to CD8^+^ αβ T cells. We did not observe any invariant NKT cells (CD3^+^TCRαβ^+^TCRα24^+^) (data not shown).

**Table 2:** Nature and percentages of FcγRIIIA^+^ (CD16) tumor-infiltrating cells among all viable cells (immune and tumor cells).

FcγRIIIA^+^ (CD16) sub-populations		Phenotype	CRC patients			
			86C	88C	90C	92C
**Total**		-	3.93	4.94	3.99	51.19
**NK cells**		CD3^−^CD56^+^	0.58	0.27	0.26	1.63
**T lymphocytes**	**αβ**	CD3^+^TCR **αβ** ^+^	0.47	0.40	0.05	0.80
	**γδ**	CD3^+^TCR **γδ** ^+^	0.18	0.05	0.01	0.03
**Monocytes/Macrophages**		CD15^−^CD11b^+^CD14^+/−^	1.47	0.23	0.78	19.16
**Granulocytes**		CD15^+^CD11b^+^CD14^−^	0.46	3.57	2.58	21.90
**Others**		–	0.77	0.42	0.31	7.66

### The Density of FcγRIIIA^+^ (CD16) Intraepithelial ADCC Effector Cells (Tumor-Associated Neutrophils and Cytotoxic T Lymphocytes) Is Higher in Tumors Than in the Normal Colonic Mucosa

The flow cytometry results were confirmed by immunohistochemistry, as we also identified the different FcγRIIIA^+^ (CD16) immune cell subtypes in the stroma (CD163^+^ tumor-associated macrophages (TAM), and tumor-associated neutrophils (TAN) as well as TILs identified morphologically) or in the epithelial compartment (TAN and some TIL but no CD163^+^ TAM). As immune cells in close contact with tumor cells can be considered as the most efficient effector cells to kill tumor cells *via* ADCC, we focused on the density of intraepithelial FcγRIIIA^+^ (CD16) immune cells, identified by flow cytometry, i.e., cytotoxic CD8^+^ T cells, NK cells and granulocytes (corresponding morphologically to TAN). As we identified morphologically 2 subpopulations of FcγRIIIA^+^ (CD16) intraepithelial immune cells, i.e., small mononuclear cells corresponding to T lymphocytes or NK cells, and granulocytes, we performed in parallel CD8 and NKp46 immunostainings. NKp46^+^ cells were very rare in tumors (around 0.03%) and mostly observed in the stroma. Thus, the majority of FcγRIIIA^+^ (CD16) mononuclear intraepithelial cells corresponded to a fraction of CD8^+^ lymphocytes rather than NK cells.

**Figure 2:**
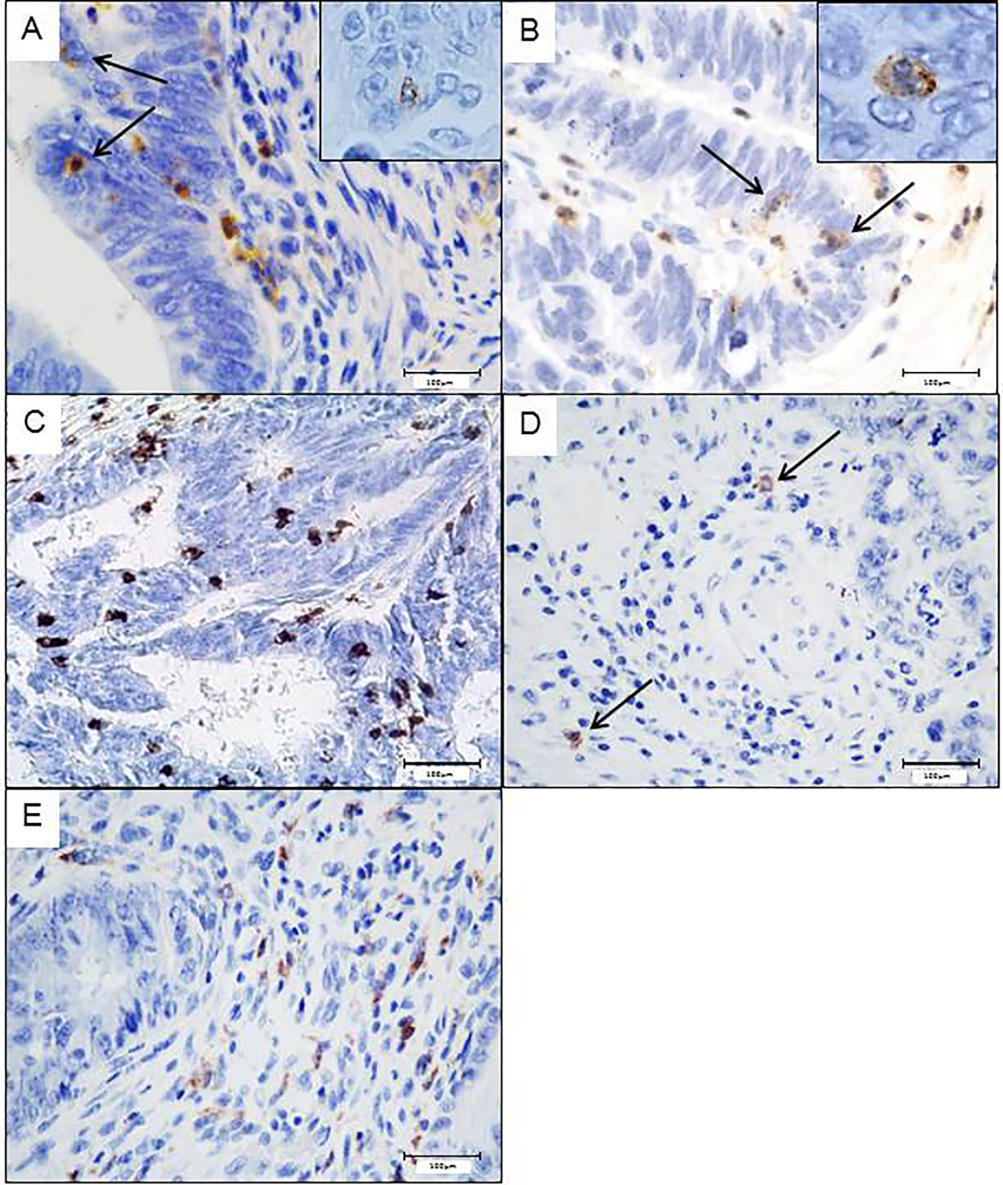
Potential intraepithelial ADCC effector immune cells in colorectal tumors assess by immunohistochemistry. Intraepithelial FcγRIIIA^+^ (CD16) lymphocytes **(A)** or neutrophils **(B)**. CD8^+^ lymphocytes in close contact with tumor cells **(C)**. Scarce Nkp46^+^Nk cells, only observed in the stroma **(D)**. CD163^+^ macrophages only found in the stroma **(E)**.

The mean percentages of intraepithelial CD8^+^ and FcγRIIIA^+^ (CD16) cells are summarized in **Table 3** and illustrated in **Figure 2**. The density of FcγRIIIA^+^ (CD16) intraepithelial effector cells was significantly higher in tumors (primary tumors or metastasis) compared with paired normal colonic mucosa (p = 0.0001). It should be noted that the percentage of each cell type, CD8^+^ or FcγRIIIA^+^ (CD16), was quite similar in the primary and paired metastatic tumors, and intraepithelial FcγRIIIA^+^ (CD16) cells were mostly neutrophils, and to a much lower degree, lymphocytes.

**Table 3:** Mean percentages of CD8^+^ and FcγRIIIA^+^ (CD16) intra-epithelial (IE) cells in normal colonic mucosa, primary tumors and metastases (Mann Whitney test).

	Normal colonic mucosa (n=11)	Primary tumor (n=39)	Metastases (n=39)	p value*	P value**
***CD8^+^ IE lymphocytes***					
*Mean ± SD*	1.66 ± 0.64	2.12 ± 2.51	1.96 ± 2.99	0.36	0.39
*Min – max*	0.82 - 2.85	0.16 - 12.91	0 - 14.9		
***FcγRIIIA^+^ (CD16) IE lymphocytes/NK cells***					
*Mean ± SD*	0.01 ± 0.03	0.44 ± 0.46	0.62 ± 0.59	0.0001	0.15
*Min - max*	0 - 0.1	0 - 2.01	0 - 2.45		
***FcγRIIIA^+^ (CD16) IE neutrophils***					
*Mean ± SD*	0.01 ± 0.03	0.75 ± 0.7	0.99 ± 1.63	0.0001	0.99
*Min - max*	0 - 0.1	0 - 2.95	0 - 9.85		

*p value between normal and tumor tissues (primary or metastase).

**p value between primary and metastatic tumors.

### The Density of FcγRIIIA^+^ (CD16) Intraepithelial TAN in Metastases Predicts a Better Objective Response to Cetuximab, Whereas Their High Density in Primary Tumors Is Associated With a Better Overall Survival

At 3 months, 15 of the 39 patients (38%) showed a partial response to cetuximab, and 24/39 (62%) were non responders, i.e., with a stable or progressive disease (13/24, 34% and 11/24, 28%, respectively) according to the RECIST criteria. Among the 15 patients who exhibited a partial response, five benefited from surgical resection of their metastases. The impact of the different intraepithelial FcγRIIIA^+^ (CD16) immune cells on the therapeutic response to cetuximab is summarized in **Table 4**. The density of intraepithelial FcγRIIIA^+^ (CD16) lymphocytes in primary tumors did not influence the clinical response to cetuximab in primary or in metastatic tumors. However, the density of intraepithelial FcγRIIIA^+^ (CD16) TAN (≥ 0.54%, 2^nd^ quartile) in metastases was significantly associated with a better response to cetuximab.

**Table 4:** Relation between the density of FcγRIIIA^+^ (CD16) immune cells involved in ADCC within tumor sites and objective response to cetuximab (p Fisher’s exact test).

	PR n = 15 (38%)	SD or PD n = 24 (62%)	Total n = 39	p value
**METASTASTIC TUMORS**			
FcγRIIIA^+^ (CD16) IE neutrophils*			
<0.54%	4 (26.7%)	15 (62.5%)	19 (48.7%)	
≥0.54	11 (73.3%)	9 (37.5%)	20 (51.3%)	0.0294
FcγRIIIA^+^ (CD16) IE lymphocytes*				
<0.50%	7 (46.7%)	12 (50%)	19 (48.7%)	
≥0.50%	8 (53.3%)	12 (50%)	20 (51.3%)	0.839
FcγRIIIA^+^ (CD16) IE cells* (total)				
<1.36%	5 (33.3%)	14 (58.3%)	19 (48.7%)	
≥1.36%	10 (66.7%)	10 (41.7%)	20 (51.3%)	0.128
**PRIMARY TUMORS**			
FcγRIIIA^+^ (CD16) IE neutrophils*				
<0.60%	8 (53.3%)	11 (45.8%)	19 (48.7%)	
≥0.60%	7 (46.7%)	13 (54.2%)	20 (51.3%)	0.6484
FcγRIIIA^+^ (CD16) lymphocytes*				
<0.38%	9 (60%)	10 (41.7%)	19 (48.7%)	
≥0.38%	6 (40%)	14 (58.3%)	20 (51.3%)	0.265
FcγRIIIA^+^ (CD16) IE cells*(total)				
<1.07%	8 (53.3%)	11 (45.8%)	19 (48.7%)	
≥1.07%	7 (46.7%)	13 (54.2%)	20 (51.3%)	0.648

*Second quartile (median); PR, partial response; SD, stable disease; PD, progression disease.

**Figure 3:**
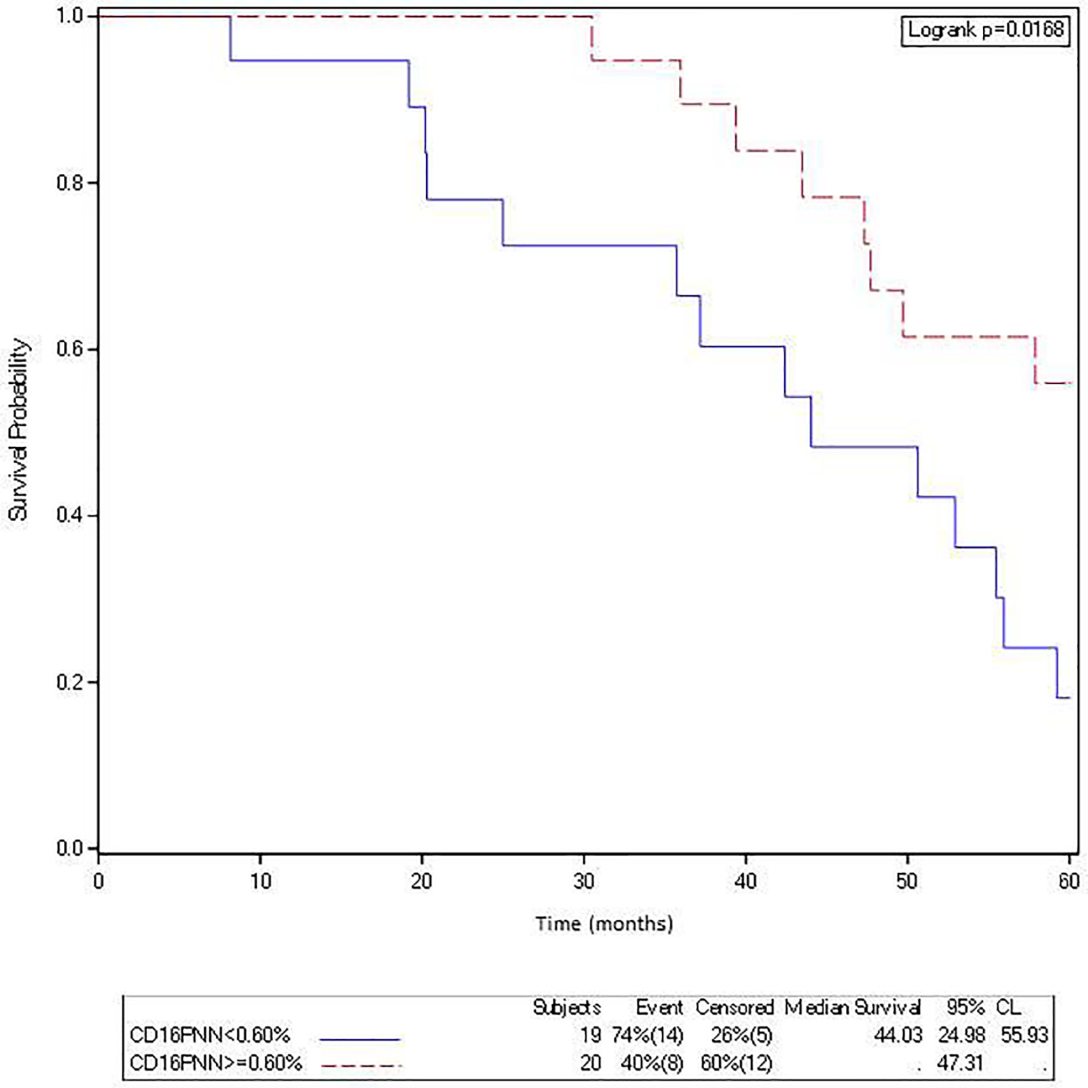
The density of FcγRIIIA^+^ (CD16) intraepithelial TAN in metastasis is associated with a better overall survival in mCRC patients treated with cetuximab. Kaplan-Meier curves depict overall survival of patients with a mCRC featuring a low (solid curve) or a high (broken curve) density of FcγRIIIA^+^ (CD16) intraepithelial TAN (second quartile).

Furthermore, a high density of FcγRIIIA^+^ (CD16) intraepithelial TAN in primary tumors (>0.6%, 2^nd^ quartile) was associated with a better OS (median not reached *vs* 44.03 months; log rank test = 0.0168) (**Figure 3**).

### The Objective Response to Cetuximab Is Not Influenced by HLA-E/β2m Overexpression by Tumor Cells or Density of CD94^+^ Intraepithelial Lymphocytes

**Figure 4:**
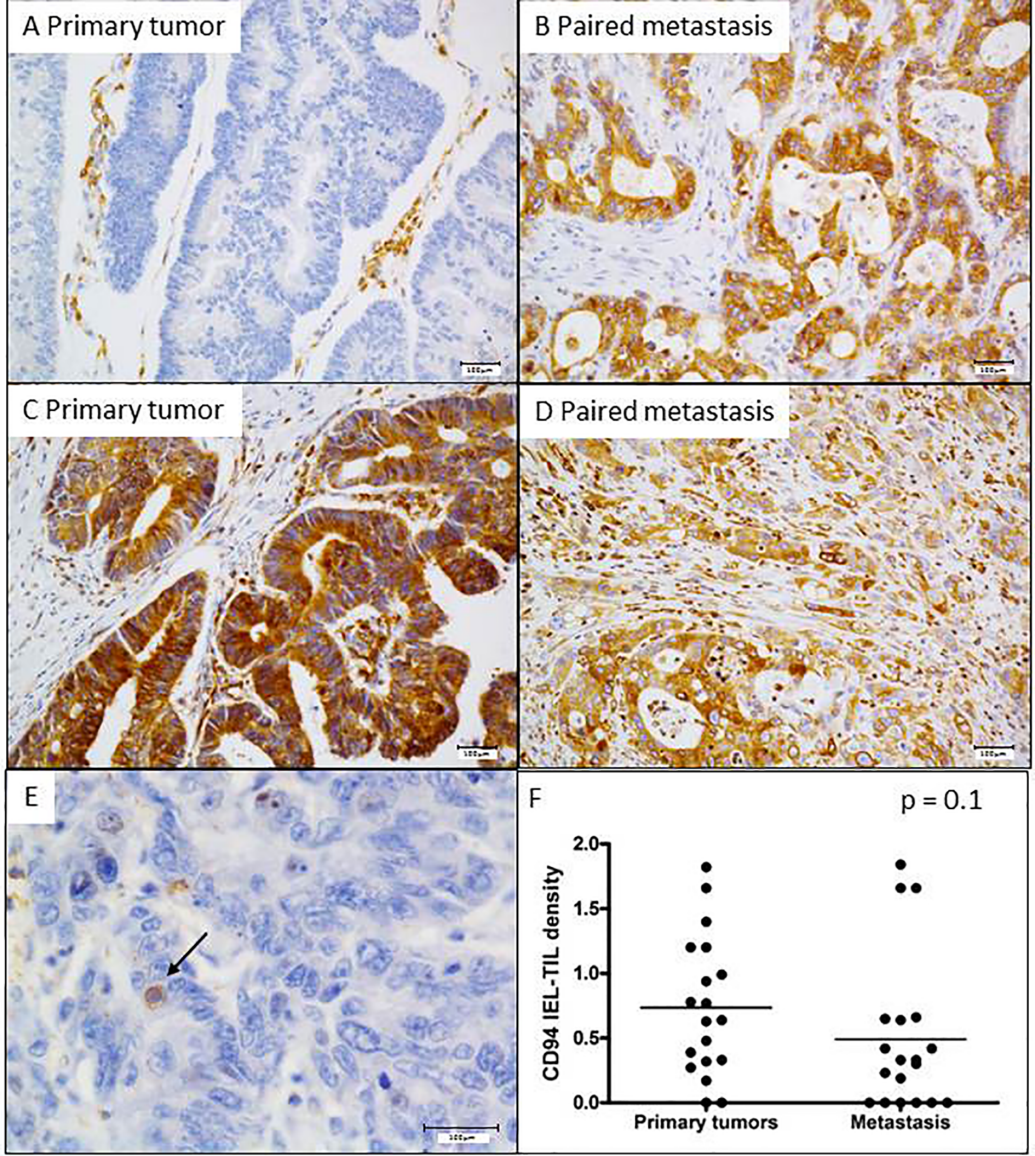
Expression profile of HLA-E in primary and metastatic tumors, and its association with intraepithelial CD94^+^ lymphocytes. Two representative cases of mCRC with distinct HLA-E overexpression profile. **(A, B)** A discordant profile of HLA-E/β2m overexpression between primary tumor (negative) and its matched metastasis (positive) and **(C, D)** a concordant case featuring HLA-E overexpression both in primary tumor and its paired metastasis. **(E)** CD94^+^ intraepithelial lymphocytes. **(F)** The density of CD94^+^ intraepithelial lymphocytes is quite similar between primary tumors and metastases (p = 0.1 Mann Whitney test).

We have previously demonstrated that about 20% of CRC aberrantly overexpressed HLA-E/β2m by tumor cells (10). This ligand is well known to interact with the CD94/NKG2A inhibitory receptor, mainly expressed by NK cells and some CD8+ T lymphocytes. Indeed, in CRC, we have shown that this receptor is preferentially expressed by CD8^+^ TILs (10, 11). Furthermore, *in vitro* data based on CRC cell lines showed that aberrant cell surface expression of HLA-E/β2m by tumor cells inhibits the cetuximab-mediated cellular cytotoxicity by NK cells. This cetuximab-induced lysis was restored by blocking the CD94/NKG2A inhibitory receptor (8), a finding suggesting that this inhibitory immune checkpoint interferes with ADCC mediated, at least, by NK cells. Thus, we correlated herein the expression profile of these potential ADCC regulatory molecules in both primary tumors and paired metastases with the objective response to cetuximab. HLA-E/β2m was preferentially overexpressed in metastatic tumors (12/39, 30.8%) compared with primary tumors (8/39 cases, 20.5%). Noticeably, HLA-E/β2m overexpression profile by tumor cells was concordant between primary and paired metastatic tumors in the majority of cases (31/39, 79.5%) but discordant in 8 (20.5%) cases. Among these discordant cases, the majority (6/8 cases) featured an overexpression of HLA-E/β2m by tumor cells in metastasis only, while only 2 cases were characterized by HLA-E/β2m overexpression in primary tumor (**Figures 4A–D**). According to the expression profile of HLA-E/β2m, the therapeutic response at 3 months was not statistically different between the 2 groups of tumors HLA-E^+^ *vs* HLA-E^−^, regardless of the tumor site considered, primary or paired metastatic (**Table 5**).

**Table 5:** Relation between HLA-E/β2m overexpression and objective response to cetuximab (p Fisher’s exact test).

	PR n = 15 (38%)	SD or PD n = 24 (62%)	Total n = 39	p value
**METASTATIC TUMORS**				
**HLA-E/β2m status**				
HLA-E^−^	10 (66.7%)	17 (70.8%)	27 (69.2%)	
HLA-E^+^	5 (33.3%)	7 (29.2%)	12 (30.8%)	1.0
**PRIMARY TUMORS**				
**HLA-E/β2m status**				
HLA-E^−^	12 (80%)	19 (79.2%)	31 (79.5%)	
HLA-E^+^	3 (20%)	5 (20.8%)	8 (20.5%)	1.0

Regarding the intraepithelial CD94^+^ immune cell density, analyzed in 19 cases, their mean percentage in primary tumors was 0.7% ± 0.5 (ranged from 0 to 1.8%) and 0.5% ± 0.5 (ranged from 0 to 1.84%) in metastases (**Figures 4E, F**). As for HLA-E expression profile, no significant relationship was observed between the density of CD94^+^ intraepithelial lymphocytes and the objective response to cetuximab at 3 months, regardless of the tumor site (primary or metastatic).

**Figure 5:**
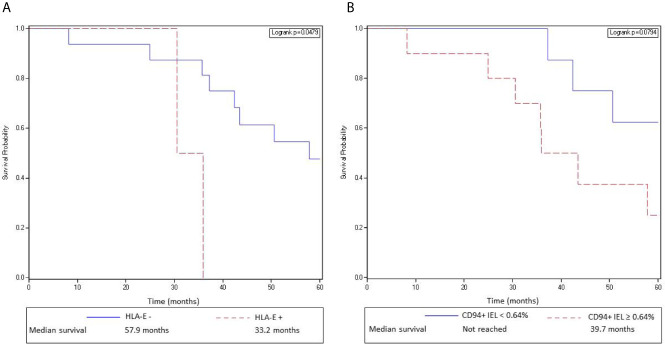
HLA-E/β2m overexpression by tumor cells and a high density of CD94^+^ intraepithelial lymphocytes in primary tumors are associated with a poor overall survival in mCRC patients. **(A)** Kaplan-Meier curves depict overall survival of patients with a mCRC without HLA-E/β2m overexpression by tumor cells (solid curve) or with HLA-E/β2m overexpression by tumor cells (broken curve). **(B)** Kaplan-Meier curves depict overall survival of patients with a mCRC containing a high (broken curve) or a low (solid line) density of CD94^+^ intraepithelial lymphocytes (second quartile).

However, as we previously demonstrated in a first cohort of 80 patients (10), we confirmed in this new independent cohort of metastatic CRC patients, that overexpression of HLA-E/β2m by tumor cells in primary tumors was significantly associated with a worse OS (median OS 35.97 months for HLA-E^+^ primary tumors *vs* 57.87 months for HLA-E^−^ primary tumors; log rank test = 0.0012) (**Figure 5A**). In the same way, a high density of CD94^+^ intraepithelial lymphocytes (>0.64%, 2^nd^ quartile) was associated with a worse overall survival compared with primary tumors containing a low density of CD94^+^ intraepithelial TIL (median 39.7 months *vs* median not reached), although the difference was not statistically significant (log rank test, p 0.079) (**Figure 5B**). In multivariate analysis taking into account the density of FcγRIIIA^+^ (CD16) intraepithelial TIL, CD8^+^ TIL (2^nd^ quartile), FcγRIIIA^+^ (CD16) intraepithelial TAN, and HLA-E overexpression, the negative impact of HLA-E overexpression in primary tumors on prognosis was confirmed (HLA-E/β2m: HR 3.9, CI [1.08;14.3], p = 0.038). This negative prognostic impact of both HLA-E/β2m overexpression and density of CD94^+^ intraepithelial lymphocytes was not observed for metastases.

## Discussion

*In situ* analysis of the tumor microenvironment emerges as an important means to understand tumor progression but also to predict therapeutic response through the close interactions between tumor cells and immune cells, especially in the promising field of immunotherapies - but not exclusively -, in order to improve their therapeutic efficacy (16). In the current study, we demonstrated for the first time, in wild-type *RAS* mCRC patients, a significant relationship between the density of FcγRIIIa^+^ (FcγRIIIA^+^ (CD16) intraepithelial TAN in close contact with tumor cells in metastases and the objective response to cetuximab. Until now, the strongest evidence supporting ADCC as a key mechanism of some monoclonal antibodies was based on studies evaluating the impact of FcγRIIIa polymorphisms on clinical response to IgG1 mAbs such as rituximab in lymphoma patients (17, 18), trastuzumab in breast carcinomas (19) or cetuximab in colorectal carcinomas (6). The fact that we demonstrated the therapeutic impact of the density of FcγRIIIA^+^ (CD16) ADCC effector cells in metastases, the main target of cetuximab in mCRC, gives a decisive evidence of the importance of this mechanism of action. Furthermore, as most of our responder patients (12/15) have received chemotherapy in combination with cetuximab, the synergistic effect of chemotherapy with cetuximab treatment to increase the efficiency of ADCC effectors can be raised. Indeed, several studies have demonstrated the major role of some chemotherapeutic drugs such as anthracyclines, cyclophosphamine, oxaliplatine, among others, to boost the anti-tumor immune response, through the induction of an immunogenic tumor cell death (20). Moreover, in the setting of ADCC, in breast adenocarcinomas, paclitaxel seems to enhance ADCC mediated by trastuzumab by rapidly recruiting ADCC effectors from blood (21). Interestingly, regarding the exact nature of infiltrating intraepithelial effector FcγRIIIA^+^ (CD16) immune cells in direct contact with tumor cells, we showed that the majority of those cells corresponded to TAN and to some T cells, but very few NK cells. Indeed, we identified only scarce NKp46^+^ NK cells, both in primary and metastatic tumors, and their density in the whole tumor (intraepithelial compartment and stroma) assessed by flow cytometry was very low. Our results are in accordance with previous studies showing the scarcity of infiltrating NK cells in CRC (11, 22, 23). Thus, these results suggest that intraepithelial NK cells, considered as the main effector cells in monoclonal antibodies-mediated tumor therapy, are not the major effector cell population in the setting of cetuximab-mediated ADCC in mCRC. Conversely, TAN constitute the main intraepithelial FcγRIIIA^+^ (CD16) ADCC effector cells, that we identified in both primary and metastatic tumors. Neutrophils are known to be potent mediators of ADCC against antibody-opsonized tumor cells, by a mechanism recently identified as trogoptosis (24). Their role in ADCC was first demonstrated *in vivo* in tumor-xenografted murine models treated with different monoclonal antibodies (24). In human, their cell killing mechanisms have not yet been completely unraveled, and more generally, the FcR-expressing cell populations responsible for the mAb-induced therapeutic effects on tumors, have not been formally identified. However, neutrophils could represent a major player of ADCC as they constitutively express FcγRIII, and this receptor is the most abundant Fcγ receptor expressed by neutrophils (25, 26). Our results underscore this assumption as we showed that these myeloid cells represent the main intraepithelial cell subtype expressing FcγRIIIA^+^ (CD16) and most importantly, their high density in metastases was associated with a better response to cetuximab. Sconnochia et al. also identified FcγRIIIA^+^ (CD16) myeloid cells in the stroma of CRC primary tumors with a phenotype (CD33^+^CD11b^+^CD11c^+^) suitable for neutrophils (23). Besides neutrophils, we also identified FcγRIIIA^+^ (CD16) and GrB^+^ (in an equal proportion) intraepithelial lymphocytes. Sconnochia et al. also identified in primary CRC tumor some FcγRIIIA^+^ (CD16) T lymphocytes without more information concerning their precise localization in the stroma or in the epithelial compartment (23). The FcγRIIIA^+^ (CD16) intraepithelial lymphocytes we identified could correspond mainly to cytotoxic CD8^+^ T cells, as suggested by our flow cytometry data. According to this hypothesis, Bjorkstrom et al. found CD8^+^ αβ T cells expressing FcγRIIIA (CD16) in the peripheral blood and in the liver of HCV-infected patients, and reported that these T cells displayed a late-stage effector phenotype and expressed high levels of perforin (27). Furthermore, it has been demonstrated that FcγRIIIA^+^ (CD16) subset of CD8^+^ T cells was able to mediate ADCC *ex vivo* and that their number increased during a virus-specific T-cell response (28). Altogether, our findings suggest that FcγRIIIA^+^ (CD16) intraepithelial TAN, as well as FcγRIIIA^+^ (CD16) intraepithelial cytotoxic T lymphocytes although found in a lower proportion than TAN, represent important ADCC effector cells in metastases in a context of cetuximab-based therapy in mCRC patients. From a clinical point of view, our results provide strong support for the use of bispecific antibody anti-EGFR/anti-FcγRIIIA as a novel strategy of antitumor immunotherapy to improve the ADCC-mediated efficiency of cetuximab in recruiting FcγRIIIA^+^ (CD16) ADCC effector cells at tumor site and offering an effective linkage between drugs and tumor target. Indeed, a phase I study has already tested a bispecific antibody (AFM13) directed against both the FcγRIIIa (CD16) and CD30, a membrane antigen strongly expressed by tumor cells, in patients with refractory or relapsed Hodgkin’s lymphoma and demonstrated its tolerability and its significant effectiveness with an overall response rate of 23% (29). One bispecific antibody targeting both FcγRIIIA (CD16) and EGF-R has yet been constructed and humanized (30) and could be tested in mCRC patients to enhance the ADCC-mediated response to cetuximab.

Interestingly, in contrast to some *in vitro* experiments based on co-cultures showing that HLA-E overexpression by CRC cell lines inhibits the cetuximab-mediated cellular cytotoxicity by NK cells *via* engagement of its inhibitory CD94/NKG2A receptor (8), we failed to demonstrate a negative impact of HLA-E/β2m overexpression by tumor cells or presence of CD94^+^immune cells on the clinical response to cetuximab. These apparent discordant results could be explained by the limitation of the *in vitro* conditions (CRC cell lines co-cultured with healthy PBMC containing the major effectors of ADCC, i.e., NK cells), which do not mimic the CRC tumor microenvironment. Indeed, the immune cell populations recruited in CRC significantly differ from those tested in these *in vitro* experiments, as we detected only scarce NK cells in line with other previous studies, but numerous FcγRIIIA^+^ (CD16) TAN which are known not to express (or scarcely) the inhibitory CD94/NKG2A receptor (31). Thus, one can hypothesize that intra-epithelial TILs, mostly corresponding to CD8^+^ TILs, could replace NK cells to inhibit the cetuximab-mediated cellular cytotoxicity as they represent the second cytotoxic cell type known to display this inhibitory receptor. However, we found an altered balance between FcγRIIIA^+^ (CD16) TAN and CD94^+^ intra-epithelial TILs in metastases (0.99% *vs* 0.5%, respectively) that could explain this lack of significant inhibitory effect on ADCC. So we can speculate that NKG2A could represent a critical player in this imbalance between TAN and CD94^+^ intraepithelial cytotoxic TILs, limiting the expansion of cytotoxic TILs without affecting TAN expansion, and thus, impairing the anti-tumor immune response mediated by those TILs.

In line with this defective anti-tumor immune response caused by exhausted cytotoxic TILs by the engagement of the CD94/NKG2A-HLA-E/β2m axis, we confirmed in this study, based on an independent cohort of metastatic CRC patients, our previous results demonstrating that overexpression of HLA-E/β2m by tumor cells (10), as well as the high density of CD94^+^ TILs (11), in primary tumors at diagnosis predict an unfavorable prognosis. Furthermore, our study, the first to explore and compare the expression profile of this new immune checkpoint in paired primary and metastatic tumors of mCRC patients, demonstrates that HLA-E/β2m is preferentially expressed by tumor cells in metastases compared with primary tumors. This differential expression of HLA-E between primary tumor and metastases was also reported by Sasaki et al. in gastric carcinoma (32). Taking into account the confirmed unfavorable prognostic impact of this new inhibitory immune checkpoint NKG2A and its ligand HLA-E/β2m in terms of OS, our results provide additional arguments to consider that HLA-E/β2m overexpression by tumor cells promotes tumor progression in CRC and facilitates the metastatic dissemination through an immune escape mechanism. They also provide convincing arguments to inhibit this new immune checkpoint with monalizumab, a humanized IgG4 anti-NKG2A antibody, as a promising new therapeutic alternative in the field of immunotherapy for mCRC patients, beyond anti-PD1 therapies.

Furthermore, we found that the density of intraepithelial FcγRIIIA^+^ (CD16) TAN in primary tumors was associated with a better prognosis in accordance with the results of Sconnochia et al. who reported the favorable prognostic impact associated with a high density of FcγRIIIA^+^ (CD16) tumor-infiltrating myeloid cells in a large series of primary CRC tumors. However, they did not report the presence of FcγRIIIA^+^ (CD16) TAN but of mainly large cells mostly corresponding to TAM (23). Our finding rather supports the tumor-suppressive function of FcγRIIIA^+^ (CD16) TAN in primary tumor at least, although in many tumors a high intratumor density of neutrophils is known to be associated with a poor prognosis, probably in line with the tumor-supportive function of some subpopulations of TAN (N2 phenotype) (33, 34). Thus, the subpopulation of FcγRIIIA^+^ (CD16) TAN we considered and counted for the first time to our knowledge, could rather represent neutrophils with N1 phenotype which exert their antitumor activity by the activation of different innate and adaptive immune cells including B and T lymphocytes, and dendritic cells (35). Indeed, it is known that NK cells and CD8^+^ T lymphocytes can be engaged in bidirectional cross-talk with neutrophils, resulting in the regulation of the adaptive immune response (36). Further studies are urgently needed to deeply explore *in situ* the complex interactions between innate and adaptive immune cells in the tumor microenvironment of CRC, owing to the identification of more accurate immune cells signatures as predictive/prognostic biomarkers.

In conclusion, we demonstrated for the first time in this study that a high density of FcγRIIIa^+^ (CD16) intraepithelial TAN in close contact with tumor cells in CRC metastases, is associated with an improved response to cetuximab. From a therapeutic point of view, our results strongly support the use of bispecific antibodies as a dual targeting strategy, both neutralizing EGFR on tumor cells and recruiting FcγRIIIa^+^ TAN in contact with tumor cells to boost the ADCC-mediated tumor cell lysis. Furthermore, the preferential overexpression of HLA-E/β2m in metastases associated with CD94^+^ TILs, provides supplementary convincing arguments to inhibit this new immune checkpoint NKG2A with monalizumab, in combination with anti- FcγRIIIA/EGFR bispecific antibodies as a promising therapeutic perspective in *RAS* wild-type mCRC patients to improve both ADCC and anti-tumor T cell responses.

## Data Availability Statement

The original contributions presented in the study are included in the article/supplementary material. Further inquiries can be directed to the corresponding author.

## Ethics Statement

Our tissue biocollection has been registered with the French Ministry for Higher Education and Research (DC-2014-2206) with approval from the ethic committee (CPP Ouest IV-Nantes). Written informed consent for participation was not required for this study in accordance with the national legislation and the institutional requirements. Except for the 4 patients included prospectively for the flow cytometry analysis who signed an informed consent.

## Author Contributions

CB and NG contributed to the study design, methodology, supervision, and manuscript draft. MM contributed to the experiments and morphological data analysis. MP contributed to the statistical analysis. CT, J-FM, FL, M-CR, and JL contributed to the tumor tissue availability and pathological annotations. SB contributed to genetic data. NJ contributed to flow cytometry analysis. TM, RF, and F-XB contributed to the clinical data availability of included patients. All authors contributed to the article and approved the submitted version.

## Funding

This work was supported by La Ligue contre le cancer, comité du Finistère (France), the 09/03 DHOS/INSERM project and the DHU Oncogreffe. This work was performed in the context of the LabEX IGO program supported by the National Research Agency *via* the investment of the future program ANR-11-LABX-0016-01.

## Conflict of Interest

The authors declare that the research was conducted in the absence of any commercial or financial relationships that could be construed as a potential conflict of interest.
